# Barriers to Partnership Working in Public Health: A Qualitative Study

**DOI:** 10.1371/journal.pone.0029536

**Published:** 2012-01-04

**Authors:** David Carlton Taylor-Robinson, Ffion Lloyd-Williams, Lois Orton, May Moonan, Martin O'Flaherty, Simon Capewell

**Affiliations:** Department of Public Health and Policy, Liverpool, United Kingdom; Yale University School of Medicine, United States of America

## Abstract

**Background:**

Public health provision in England is undergoing dramatic changes. Currently established partnerships are thus likely to be significantly disrupted by the radical reforms outlined in the Public Health White Paper. We therefore explored the process of partnership working in public health, in order to better understand the potential opportunities and threats associated with the proposed changes.

**Methodology/Principal Findings:**

70 participants took part in an in-depth qualitative study involving 40 semi-structured interviews and three focus group discussions. Participants were senior and middle grade public health decision makers working in Primary Care Trusts, Local Authorities, Department of Health, academia, General Practice and Hospital Trusts and the third sector in England. Despite mature arrangements for partnership working in many areas, and much support for joint working in principle, many important barriers exist. These include cultural issues such as a lack of shared values and language, the inherent complexity of intersectoral collaboration for public health, and macro issues including political and resource constraints. There is particular uncertainty and anxiety about the future of joint working relating to the availability and distribution of scarce and diminishing financial resources. There is also the concern that existing effective collaborative networks may be completely disrupted as the proposed changes unfold. The extent to which the proposed reforms might mitigate or potentiate these issues remains unclear. However the threats currently remain more salient than opportunities.

**Conclusions:**

The current re-organisation of public health offers real opportunity to address some of the barriers to partnership working identified in this study. However, significant threats exist. These include the breakup of established networks, and the risk of cost cutting on effective public health interventions.

## Introduction

The public health function in England is facing dramatic change. Until recently Primary Care Trusts (PCTs) were responsible for the local public health function in England. These primary care bodies supported local groups of general practitioners (GPs), and acted as the main commissioning and primary care development organizations. The public health function was also devolved to these organisations, and in order to aid partnership working in local areas, many PCT Directors of Public Health were jointly appointed between PCTs and Local Authorities (LAs) – these are the organizations responsible for a range of local services including housing, social services and urban regeneration [1]. Partnership working between PCTs and LAs has been facilitated through the development of Local Strategic Partnerships (LSPs), which take the form of partnerships between public, private and third sector organisations with the aim of creating a framework within which local partners can work together more effectively to secure the wellbeing of their area. These partnerships agree appropriate local targets (Local Area Agreements), informed by jointly undertaken needs assessments (Joint Strategic Needs Assessments) [2].

These currently established partnerships of organisations working to improve health are likely to be significantly disrupted by the reforms outlined in the Public Health White Paper, Healthy Lives, Healthy People [3], which were heralded in the NHS white paper “Equity and Excellence: Liberating the NHS” [4]. This signifies a major change in the way in which public health services will be provided and delivered. Most significantly, the White Paper describes the creation of a new, integrated, national public health service, Public Health England, with local public health teams returning to local authorities. The new local authorities will have increased responsibilities to coordinate overall health policy for a geographic area, joining together the work of local government, the NHS and the new National Public Health service [5].

Partnership working is widely advocated in order to implement strategies to influence the wider determinants of health and health inequalities, and thus secure population health improvement [5], [6], [7], [8], [9], [10], [11], [12], [13]. Though partnership working as applied to public health is a difficult concept to define, and the term can be used to mean a multitude of things [2], [14], the motivation for partnership working is easier to pin down. Hunter et al describe this as the “the consequence of recognition that no single agency can possibly embrace all the elements that go to contribute to a policy problem or its solution.” Effective public health provision in the UK has often been characterised by this type of strategic partnering. Furthermore constructive collaborative relationships between local authorities and health service partners have been widely established in the last decade [9], [15], [16].

A recent systematic review highlighted the limited evidence base around partnership working in public health [7]. Furthermore another large postal study of partnership working identified the need for further studies exploring causes of partnership failure, particularly with reference to local authority partners [17]. In order to better understand the potential opportunities and threats associated with the proposed changes in public health, we undertook a qualitative study of public health policy makers aiming to explore attitudes to partnership working arrangements in public health, with a particular focus on barriers to successful partnership working. We explore the relationships (both formal and informal) amongst individuals and groups from a variety of backgrounds involved in partnership working around cardio-vascular disease. This was undertaken as part of a larger study exploring the use of evidence in public health decision-making.

## Methods

### Ethics

We sought advice regarding ethical approval from the appropriate committee (North West Research Ethics Committee), and were advised that the project did not require formal review under the terms of guidance for NHS research ethics committees in the UK. This was due to the nature of the sample - NHS employees and policy makers, rather than patients. All participants gave informed consent to take part in the study.

### Design

An in-depth qualitative design, employing interviews and focus group discussions and informed by ethnography, was chosen to allow exploration of the meanings and perceptions of participants regarding partnership working against the backdrop of the overall context of decision making in public health policy. It was conducted by a multidisciplinary team with varied backgrounds and experience, including: medical anthropology (LO: main researcher), clinical epidemiology (SC and MOF) and public health (DTR, FLW and MM).

### Participants and setting

Research participants were involved in decision making around CVD at a local, regional or national level in the UK. Coronary heart disease is one of the most important contributors to mortality and morbidity in the UK, with well-established organisational frameworks for partnership working and decision-making. Some of these partnerships are specific to CVD, such as the cardiac networks which have a clinical focus [18], and the Heart of Mersey initiative which has a prevention focus [19]. In addition individuals working on CVD sit on a variety of cross-cutting groups working to promote health more broadly e.g. to promote exercise and health eating in children. In this respect individuals working on cardiovascular disease prevention are well placed to comment on the partnership working agenda. The initial sampling frame for the interviews comprised 58 individuals involved in the public health policy and decision-making process around CVD in a range of organisations in England. Two distinct strategies were used to generate this diverse pool of participants: Firstly, a list of known individuals involved in the policy and decision-making process was drawn up on the basis of existing professional networks. Secondly, a purposive sampling strategy explicitly sought to include individuals from organisational types that were under-represented in the initial list of known policy/decision makers' organisations. Subsequently we used theoretical sampling, and we sough participants strategically to refine and test the developing analysis. These lists were then combined to generate the final sampling frame. A recruitment letter was sent to every person on the list – this gave background details and invited the recipient to participate in the study. The interviews took place from November 2009, through to the end of 2010, spanning key events influencing the NHS and healthcare in England [20]. These include the general election in May 2010 and the subsequent publication of the NHS white paper in July 2010 [4], followed by the publication of the public health white paper in November 2010 [3].

### Interviews

In depth semi-structured interviews were the main method of data collection. Prior to the interview, participants received an introductory letter, which provided further information about the consultation process, and written informed consent to participate was taken. A topic guide was developed, the content of which evolved as data analysis progressed and the research focus became clearer. The topic guide covered open ended questions about the policy making process; the nature of decision-making across organisations; the use of evidence; and explored barriers and facilitating factors influencing partnership working and the use of evidence across organisations. Partnership working around CVD was used as an initial example, but the key consideration influencing the direction of each interview was the participant's answers to questions in terms of their individual experiences. The interviews were conducted by LO and MM. Table 1 outlines the roles of the interview participants.

**Table 1:** Interview participants.

Role	Number of participants
CVD commissioners	7
Public Health practitioners	4
Data analysts	2
PCT Researcher	1
PCT knowledge manager	1
Local authority employees	2
Joint LA/PCT roles	3
GP commissioner	1
Public health academics	7
NHS consultants	7
National guideline manager	1
Lay member of a guideline development group	1
Civil servant	1
Third sector staff working on CVD prevention	2
**Total**	**40**

### Focus groups

The three focus group discussions were conducted in order to explore key emergent themes from the individual interviews and to address some of the discrepancies and gaps in the interview data [21]. For each focus group we aimed to recruit between six and 10 participants. After taking written informed consent, participants were presented with a summary of the interview findings and were encouraged to develop and reject the ideas presented to them, providing a method of respondent validation [22]. We took a pragmatic approach to recruitment to the focus groups, and made use of public health events where decision makers were already due to congregate. The first focus group included participants who were involved in decision-making around CVD in one Primary Care Trust and were therefore known to each other. The participants in the first focus group had also taken part in individual interviews, and we were thus able to undertake a process of member checking with this group. The subsequent focus groups were used to test and develop themes further. All participants attending a particular meeting at the PCT were invited to take part in the focus group. The second included participants at a public health conference, and targeted individuals involved in CVD policy were invited to attend the focus group. The third was conducted at a public health training event, where individuals had the option to join in the focus group discussion as a parallel session.

### Analysis

The analysis sought to identify associations between themes and to carry out an in-depth exploration of the emergent findings. All interviews and the focus group discussion were recorded electronically and analysed using NVivo software for qualitative data analysis (version 7). The analysis used techniques drawn from the constant comparative method [23]. Transcripts were coded line-by-line based on the meanings, perspectives, and actions which they represented, and for contextual factors in their generation. A subset of 25 per cent of transcripts were double coded by two members of the research team, disagreements and insights were discussed and alternative interpretations were incorporated in the analysis. The analysis was further tested during discussions with colleagues, through meetings of the project steering group, and in the focus group discussions. Through this process, the aim was to identify the “big ideas” (or themes) that were grounded in the data [24].

### Validation

DTR, MM and FLW coded a subset of 25 per cent of transcripts as a check to ensure high levels of inter-researcher consistency in analysis. Disagreements and insights were discussed and alternative interpretations were incorporated in the developing analysis [25]. The focus groups were then used as a further source of data for member checking, triangulation, and testing of theories.

## Results

Seventy-nine senior and middle grade public health decision makers in CVD from across England were approached to take part in an interview. Thirty-nine declined and 40 participated (table 1). The first focus group included seven informants, all of whom had also taken part in an interview. They included: three consultant cardiologists; and two public health consultants, a public health doctor, and a knowledge manager from one PCT. The second focus group included 10 new informants (not included in interviews), all with an academic or practical interest in public health and the prevention of CVD. The third focus group included 20 regional decision makers working in public health (not included in interviews). Overall 70 participants took part in the study in 40 semi-structured interviews and three focus group discussions. Most interviews lasted about 45 minutes, ranging from 20 minutes to one hour and fifteen minutes. The first focus group lasted 70 minutes; and the second and third 60 minutes. The main findings from interviews and focus group discussions are presented together, below.

### Examples of partnership working

Numerous examples were cited of areas of joint working between the traditional health sector, local authorities, academia and the third sector (text box S1).

### Benefits of partnership working

In general positive views emerged in relation to working across sectors, and practical examples were provided where mature organisational structures and working groups have been established, around issues with a specific health remit. An important theme was the need for, and clear benefits associated with joint working, in terms of influencing the broader determinants of health, and addressing health inequalities. These respondents expand on these themes:

*We have joint commissioning around…well older people. You have got obviously health issues but you have also got social care issues and very often it's the link between the two that gives the best results rather than behaving in silos. It's always been you know a big problem in us actually delivering holistic health care that you don't make the proper links with other agencies that actually make it better for patients*

*P25 – cardiac programme manager*

*We have a number of working groups across key areas, co-chaired by someone from health or someone from local authority or someone from another sector…so, we try and forward the, if you like, the preventative end of the public health agenda and to promote health and well-being. And, clearly to try and address the issues of health inequalities.*

*P35 – NHS/LA joint appointment*

### Factors that facilitate partnership working

It was perceived important to properly understand the organisations that one was attempting to influence, and to do this required inside knowledge. In this respect, joint appointments were felt to be particularly valuable. This respondent outlines this view clearly:

*I think where your host organisation is has a huge impact on your influence. And from my perspective, understanding how the political system works from the inside is so much, so much easier to influence from the inside than it is from the NHS looking in.*

*P38 – joint LA/PCT worker (health and social care)*

Joint appointments between the NHS and LA were also felt to allow the profile of preventative approaches to be more effectively raised within NHS structures.

Partnership networks, both formal and informal have been established in many areas. Key to the success of these groups are clear aims and objectives, and frameworks for decision making and implementation of strategies, as outlined by this respondent:

*I mean we are now what's known as a Section 75, which is a partnership agreement with the City Council. So we have an arrangement now where there is delegated functions and therefore delegated decision making…So that we get that joined up partnership working arrangement.*

*P38 – joint LA/PCT worker (health and social care)*

### Perceived barriers to partnership working

Significant barriers to joint working were identified, including the inherent complexity of influencing the determinants of health across sectors. These are summarised in text box S2, and described in more detail below.

### Barriers – dealing with complexity

Dealing with the complexity of public health decision-making and primary prevention to influence the broader determinants of health was felt by many participants to be very challenging. One theme that emerged was the perception of limited influence at the local level. Decisions were felt to be taken at a national (government) or international (EU) level, with population-wide primary prevention thought only to be effectively tackled through national or international efforts. There was the sense that this could lead to a feeling of being overwhelmed by the complexity of the task, as expressed by this first respondent, referring to a group considering the evidence base for cardiovascular disease policies:

*It's a nightmare, god with cardiovascular (laughs) well where do you stop? They look at government policy, national government, regional government, European government, you know economic policy, different types of political and social organisations so you know the breadth of it is immense.*

*P23 – public health academic*

*Trying to address the primary prevention agenda, it's obvious that a large part of the agenda has to be addressed at national or EU levels.*

*S1 – CFGD*

Underpinning the issue of complexity were concerns about the difficulty of tracking inputs and outputs over long time frames, using imperfect data, and imperfect tools. This compounded the substantial challenge of sustaining arguments for public health interventions in the face of limited resources. Some participants were concerned about the difficulties of measuring outcomes, and of ensuring that health was considered an important outcome across sectors, where partnership working may also be focussing on other outcomes, such as employment, resident satisfaction or educational performance measures. One participant suggested that public health professionals needed to become more adept at highlighting the health effects of interventions and policies that are under the jurisdiction of other sectors:

*I think the other issue, you're possibly touching on this now, is how can we politicise public health as a key outcome that needs to be considered in other policy areas, whether it be transport, whether it be agriculture etc.*

*P107 – CFGD*

But one participant suggested that integrating health into this broader agenda was made more challenging by the perceived lack of a theoretical framework for action across sectors. Speaking in the context of implementing recommendations to increase physical activity, one respondent outlined this issue:

*What I never really felt we received sufficiently was a good theoretical framework on which to hang this…how the implementation of the recommendations might be parcelled up amongst sectors or different sort of user groups…*

*P23 – public health academic*

### Barriers – Cultural issues

Silo working persists within the current system of partnership working. Prioritisation of different outcomes across sectors was seen as one of the issues driving this, with the acute sector perceived to be primarily interested in short term health outcomes, local authorities interested in social outcomes, and the PCTs trapped in a vacuum between the two. A cardiologist working on a CVD prevention programme suggested that this silo working meant that public health professionals based in PCTs had limited influence:

*You know you're well intentioned (Public Health) and you know what to do. But you neither have the medical or NHS connections to enable you to do it nor the social and political connections in order to implement what you want to do. I think that's quite significant in my mind.*

*FGD – consultant cardiologist*

Perhaps symptomatic of this silo working was the perceived language barrier between different sectors, and the use of acronyms and impenetrable specialist terminology, which was viewed as a barrier to effective communication:

*When you speak to local authority representatives, it's erm, it's like talking to an alien. And they feel the same to us because we use acronyms in the NHS like QUIPP and DOUGIE all that sort of stuff. So we're trying to get a foot in both camps really as a starter for six*

*P05 – public health academic*

Contributing to the language barrier was the difficulty of reconciling what is good evidence for an inter-sectoral intervention, because different sectors value evidence in different ways. For instance, town planners trusted CABE guidelines (Commission for Architecture and the Built Environment), whereas PCT staff frequently cited NICE and Cochrane. There was a perceived lack of evidence based guidance that spanned sectors. A key issue raised was the identification and packaging of this evidence to persuade policy makers to invest in upstream preventative interventions. Arguments based on economic effectiveness were perceived to be particularly influential.

*An exceptionally robust evidence base (is needed) to show that this is robbing Peter to pay Paul effectively in terms of making up cuts at this moment, which will result in a much greater burden on the Health Service.*

*S8 – CFGD*

In the context of a sustainable transport intervention, this participant outlined how the public health community lost the argument, partly because they could not bring to bear the key economic arguments:

*So they say like this road will improve congestion and will reduce journey times and we can put a value on people's time…But they weren't very good at valuing the health benefits, and if we're talking about building bypasses compared to getting people out of cars into walking and cycling then that's a major, major part of the benefit*

*P32 – NHS/LA joint appointment)*

Some participants felts that this type of policy relevant evidence was potentially available, but that public health advocates were not learning lessons from other settings.

*We need to show how places such as Denmark or New York State have pushed this [trans-fat ban] through in terms of legislation and what evidence they used in terms of persuading policy makers to change their opinion on this….this sort of approach needs to be replicated*

*S4 – CFGD*

The NICE physical environment guidance was cited as an attempt to develop cross sector guidance. However, one of the problems with this was the dissemination and implementation of guidance across sectors:

*There was no implementation (of the NICE guidance) to the professional bodies who were expected to implement the health principals we want…So town planning, urban design, architects and transport professionals who work in local authorities…there is no ability to transfer what we've done from a heath sector perspective into their professional bodies and practices*

*P23 – public health academic*

Further complicating the relationships between sectors was the issue of jurisdiction over particular outcomes and the perceived territorial demarcation between organisations. It was perceived that the health sector could be seen to be speaking out of line when making recommendations that could affect other organisations, and that this could lead to politically difficult negotiations:

*The interesting thing was within government, the number of agencies who got pretty upset about NICE making certain recommendations. For example, one of was about CAP (Common Agricultural Policy) – DEFRA (Department for Environment, Food and Rural Affairs) went bananas….*they got all sorts of comments back saying ‘Well you're the NHS, why are you telling the food industry what to do?’

*P16 – public health academic*

A key theme that emerged was the systematic undervaluing of preventative interventions, especially in the context of limited resources. Some suggested there was a culture of paying lip service to prevention, leaving preventative initiatives particularly vulnerable to cuts. Furthermore some felt that this was still the case, despite evidence to suggest that preventative interventions could be cost saving:

*But in the grand scheme of things, in their list of priorities when cuts are being made and money is very scarce and resources are scarce, prevention isn't gonna be something that's high on the agenda*

*S127 – CFGD*

*Everybody kind of pays lip service to it and says prevention is even more important in the current climate, yet at the same time it seems to be the most vulnerable element of the health budget in some respects.*

*S119 – CFGD*

### Barriers –Macro level influences

There was the recognition by some that intersectoral working for public health is highly political, and there were powerful lobby groups also trying to exert influence. Speaking about attempts to limit advertising of unhealthy foods to children, and to regulate vending machines in schools, one participant suggested:

*There are a lot of competing interests at political level. I think the health lobby are pretty naive and pretty inefficient at getting, you know that kind of political support*

*P23 – public health academic*

Some felt that the current government's focus on behavioural “nudging” - encouraging individuals to take responsibility for their own health behaviours, rather than the government taking the lead in creating healthy environments - was the result of industry influence, and was viewed with skepticism by public health professionals in our study:

*Somehow the state was [seen to be] too involved in the past, when I saw it as barely involved at all, and that these are matters of individual responsibility when individual responsibility is absolutely no safeguard against an ecological setting which is designed to overcome individual responsibility.*

*(P03, public health academic)*

Public pressure, expressed by local constituents, was also cited as being particularly important, and this tended to focus on issues of healthcare access, rather than more upstream issues. Reasons cited for this included the fact that debates around acute services tend to be more high profile, and more likely to “hit the headlines”.

*Politicians listen to their constituents as opposed to the public health professionals*

*S122 - FGD*

*Secondary care services, acute services, tend to dominate the agenda both in the public mind, politically and within Health and Social Care.*

*P20 - Public health consultant*

As outlined above, economic considerations were of paramount concern in the current climate, and it is clear that many feel that prevention is particularly vulnerable, especially given the longer timescales needed to demonstrate benefits:

*Interventions of that type at this moment in cash-strapped times are seen as luxury interventions, and the focus has been on operational issues in cash-strapped times*

*S91 - CFGD*

So overall there was the perception that the capacity to shift the balance towards partnership working for health was constrained by a plethora of high level influences. This participant describes the dynamic nature of this complex system:

*So we see a very complicated picture, sort of a dance of different interests and different issues at the moment shaping the messiness of policy making…*

*(P05, public health academic)*

### The future

In principle the re-location of public health to a local authority setting was felt to be a positive step by some. This participant expressed optimism, suggesting that this move represents a homecoming for the public health function:

*So, I think there, there is a lot of coming back together again because planning actually came out of public health and housing really. So that was it's original function – I think it kept the links with housing quite well but it lost it with public health. So, I can only see this, you know, this, er, working together as being a good thing really.*

*P37 – LA employee*

Others saw new opportunities for decision makers at all levels to raise the profile of population-wide primary prevention at a national level, given that the new coalition government has expressed a renewed commitment to addressing health inequalities. However, there was the concern that this will only be met if all sections of government act in a coherent way to address the underlying determinants of health and health inequalities:

*There are a lot of issues that reflect on health that require cross-Government working – I think that will be an opportunity with this new administration.*

*P36 - public health academic*

A key concern was that yet another re-organisation would lead to the breakup of established partnerships that have developed over a number of years. In the context of a healthy eating project, involving convenience store shops, one participant expressed concern that projects were not being re-commissioned, just at the point at which they were beginning to deliver concrete outcomes.

*It's taken 10 years to get to this point, and many other areas probably won't have had the chance to see the range [of effects]*

*P30 – LA employee*

Participants also suggested that many of the concerns about silo working could equally apply to any new organisational structures. One participant suggested that there was a tendency for local authorities to plan on the basis of small areas, such as neighbourhoods, and that this mitigated against a joined up population approach. Furthermore, there was concern that partnership working on public health issues with GP consortia would be challenging, due to the lack of co-terminosity and governance structures.

*I think there will also be challenges because certainly where I work, we're actually working at neighbourhood level so you know, we're gonna be working in even smaller silos*

*S89 – CFGD*

*We also worried about the outcome frameworks that are coming out. Because you're gonna have a different one for the Local Authority and Public Health and a separate one for GP Consortiums - there needs to be targets which are the same in both.*

*S98 – CFGD*

## Discussion

We undertook a qualitative study to explore attitudes to partnership working to improve public health, in the context of the proposed changes to the NHS in England. We found that mature arrangements for partnership working existed in many areas, and there was much support for joint working in principle. However, many barriers exist. These include the inherent complexity of intersectoral collaboration for public health; cultural issues such as a lack of shared values and language; and macro issues such as political and resource constraints. The extent to which the proposed reforms will mitigate or potentiate these issues is unclear, but there are clear threats, while potential opportunities remain challenging.

This study corroborates the findings that have emerged in other studies of partnership working. In a recent systematic review of quantitative and qualitative studies, Smith et al explore the evidence relating to the health impacts of public health partnerships. They conclude that although many studies report positive attitudes to partnership working, very little is known about actual health impacts of public health partnerships as they have not yet been rigorously evaluated [7]. In our study, a similar enthusiasm was expressed for partnership working, alongside the identification of important barriers. Hunter et al identify some similar barriers to partnership working including participants in strategic partnerships being overwhelmed by the size of the agenda; difficulties of sustaining governance arrangements in the context of re-organisations; lack of trust; resource constraints; and tokenistic partnering [8], [9], [13]. Pettricrew et al. conducted in-depth interviews with senior policy makers in Scotland and identified barriers to integrated policy making, including: a lack of political leadership and ministerial engagement; insufficient rewarding of intersectoral work; the persisting influence of traditional departmental structures and boundaries, and scarcity of resources [6]. Griffiths et al conducted a questionnaire survey of public health consultants and specialists and identified concerns around: the inherent difficulties of working across geographic, structural and professional boundaries; and lack of a clear structure in which to work [26]. Bauld et al also highlight the need for shared governance structures to be embedded in local systems, at a range of levels across organisations in order to facilitate effective joint working [15]. The participants in our study highlight the importance of clear lines of communication, and governance structures, and the difficulties of “getting under the skin” of other organisational structures. The disruptive effect of frequent re-organisations on morale and public health capacity has been noted in other studies, as has the critical issue of resource security [11], [15], [26], [27], [28]. Griffiths et al found “there was real concern that without adequate support, funding and increased capacity, public health would not realise its full potential.” [26]. In their study of partnership working in Scotland, Richie et al describe how a lack of staff continuity within partnerships can be particularly disruptive, through the loss of individuals with particular technical expertise, but more importantly as a result of the erosion of the shared theoretical positions, vision and understanding which appear essential for successful outcomes [29].

The current economic crisis has pushed cost-cutting to the top of the agenda across government departments [27]. Worryingly, in our study, there was the clear feeling that preventative initiatives were particularly at threat, even if it were possible to demonstrate that these would be cost saving in the longer term. As Hunter [[8], p145] states “we have failed to put health before health care”. Furthermore Blackman et al describe the issue as a “wicked problem” for which there are no clear solutions [30]. As in our study, Marks et al also identify concerns around protectionism of departmental budgets across partnerships [13]. This is of particular concern to public health practitioners preparing for the proposed move to local authorities. Although there are plans for a ring fenced public health budget, in some way weighted for inequalities, this must be considered against the backdrop of widespread cuts to local authority budgets across the country which are greater in more deprived areas [31], [32]. Furthermore there is ongoing debate about the precise amount of public health funding that will be required, how this should be allocated, and whether ring-fencing is appropriate in the context of the current government's proposed more towards place-based budgets [33].

Other studies have highlighted the potential clash of cultures between the health sector and local authorities [11], [34]. In terms of bridging these cultural differences, Jones et al's large postal survey of attitudes to intersectoral working found that trust and leadership were the most important predictors of sustainable partnerships [17]. Integrative leadership skills are particularly important in multi-agency partnerships where it is sometimes difficult to identify who is in charge. These skills include understanding the social and political contexts, communicating and sharing a vision and implementing policy decisions across organisations [17]. In our study, there was clear concern that the networks of trust that have developed over time will be disrupted by the current re-organisation process. In their recent study Hunter et al describe the “goodwill, trust, and passion shared in achieving better public health outcomes” as the principal features that drive partnerships forward [2].

As we move into a new era of public health provision, with local authorities being the main public health provider, our data provide some insights and possible directions for future partnership working. Opportunities exist to harness the passionate belief in joined-up action across sectors to improve public health outcomes. Partnership working was highly valued by most of the participants in this study, and seen as the only way to address the public health challenges that we face. However, there were significant concerns that the already established collaborative networks may be completely disrupted as the proposed changes unfold, in parallel to the ongoing attrition of the public health workforce [35], [36], [37]. The proposed changes offer the opportunity to address some of the cultural barriers that have been identified in this study (text box S2). While the context of this study is the public health system in England, with particular reference to the current changes, our findings are relevant to other health systems grappling with issues of fostering partnership working whilst undertaking organizational change. Many of the issues raised are generic and encountered whenever inter-sectoral co-operation is required [11], [16]. For instance, the language barrier, and lack of shared commissioning, management and governance structures identified by participants in this study. These could be addressed if the new local authority structures facilitate a more integrated approach, and avoid “silo-based” practice. There is, however, the danger expressed by some participants in this study, that the proposed separation of public health from the NHS, both financially and organisationally, will mean the NHS no longer sees ‘health’ as its responsibility, only health care, and it will therefore focus solely on treating ill health, not preventing it [13]. Perhaps of more concern are the barriers identified in our study relating to complexity and macro issues. There is clear uncertainty and anxiety about the future of joint working relating to the availability and distribution of scarce and diminishing financial resources. Furthermore, achieving a truly evidence-informed approach to public health policy across sectors seems a distant prospect [13], [30].

This study has various limitations. Although we have cast the net widely in terms of participants, the sample may not represent the full spectrum of views with regard to the multiple stakeholders involved in partnership working. This study has focused on the relationship between local authorities and the health sector, because local authorities in the UK have a key role in influencing the broader determinants of health, through spending on areas such as children's services, adult social care, and planning and regeneration [38]. Though this relationship is of key importance for public health action on the broader determinants of health, we acknowledge that there are other relevant stakeholders that have not been included in this study, including the private sector. It is also difficult to accurately represent the views of subgroups within the sample, and explore differences between groups. This is because of the large number of professional groups and organisational levels involved in inter-sectoral decision-making. However, our main aim was to identify common themes that arose across the whole sample.

Public health provision in the UK during the last decade has been characterised by strategic partnering. However, potentially important barriers to partnership working in the existing public health structure include a lack of shared language, commissioning, and governance frameworks, and differences in the use of evidence across sectors. The relocation of public health to local authorities as part of the reorganisation of public health provision in England has the potential to facilitate more effective joint working in terms of the relationship between the bodies that influence the “social determinants of health”. There is also a real opportunity to address some of the barriers identified in this study as part of the re-organisation of public health. However, there is also the risk that lessons are not learned, and that similar problems are replicated in the new system. Significant threats exist. At the micro level these include the breakup of established networks, and the systematic undervaluing of preventative interventions. At the macro level the climate of financial insecurity, and the influence of broader political and corporate interests are seen as substantial barriers to progress.

## Supporting Information

Text Box S1 **Examples of partnership working.**
(TIFF)

Text Box S2 **Perceived barriers to partnership working.**
(TIFF)
