# Supplementary figures and images for: Barriers to Partnership Working in Public Health: A Qualitative Study
Source: PLoS One. 2012 Jan 4;7(1):e29536. doi: 10.1371/journal.pone.0029536 (PMC3251584; doi:10.1371/journal.pone.0029536)

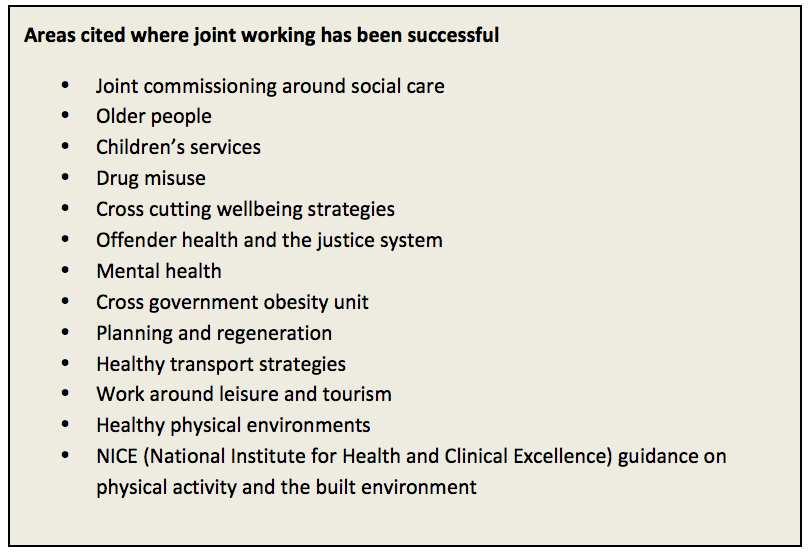

Supplement: Text Box S1 — Examples of partnership working. (TIFF) [file pone.0029536.s001.tiff]

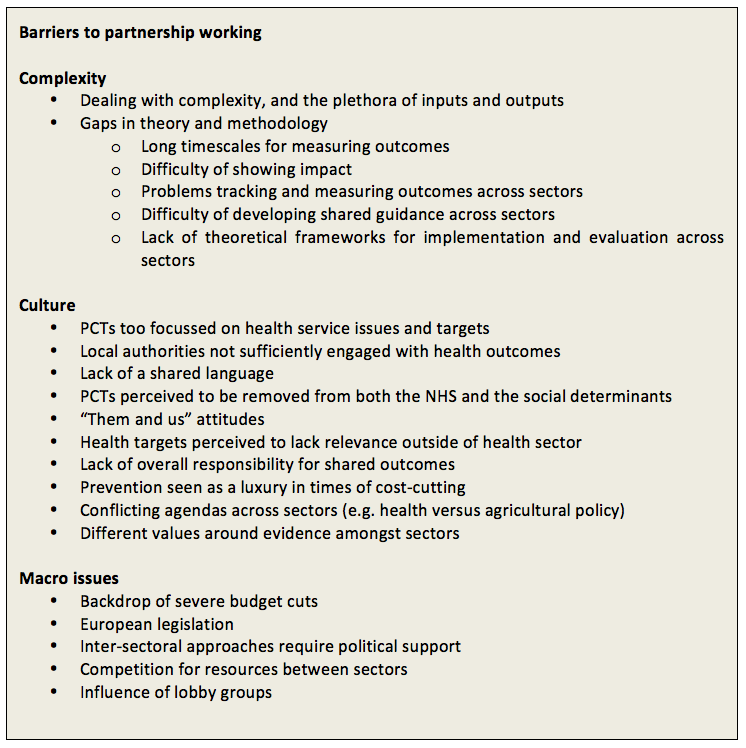

Supplement: Text Box S2 — Perceived barriers to partnership working. (TIFF) [file pone.0029536.s002.tiff]
